# Contributions of the anion exchangers SLC26A1, SLC26A2, SLC26A3, and SLC26A6 to intestinal oxalate transport in human Caco2‐BBE cells

**DOI:** 10.14814/phy2.71125

**Published:** 2026-10-09

**Authors:** Khalid A. Abdelhalim, Donna Arvans, Yanzhe Wang, Mohamed Elfatih H. Bashir, Hatim A. Hassan

**Affiliations:** ^1^ Division of Nephrology and Hypertension Mayo Clinic Rochester Minnesota USA; ^2^ University of Chicago Pritzker School of Medicine Chicago Illinois USA; ^3^ Department of Physiology and Biomedical Engineering Mayo Clinic Rochester Minnesota USA; ^4^ Present address: Portal Innovations, ClostraBio, Inc Chicago Illinois USA

**Keywords:** anion exchangers SLC26A1, SLC26A2, SLC26A3, and SLC26A6, intestinal oxalate transport, kidney stones, relevance to hyperoxaluria and hyperoxalemia, siRNA knockdown

## Abstract

Kidney stones affect ~1 in 5 men and 1 in 11 women, with 70%–80% being composed of calcium oxalate. Knowing the contributions of anion exchangers SLC26A2 (A2), A3, and A6 to apical and A1 to basolateral oxalate transport in human intestinal Caco2‐BBE (C2) cells is important since enhancing intestinal oxalate secretion (A6/2/?A1) and reducing intestinal oxalate absorption (A3) are potential therapeutic targets. We previously showed A6 contributes > A2 to oxalate transport in C2 cells. Here, we report the contributions of combined A2+A6, A1, and A3 to oxalate transport in C2 cells using siRNA knockdown. A2 (74% reduced mRNA expression), A6 (64% reduced protein abundance), and A2+A6 (combined knockdown utilizing same A2 or A6 C2 silencing batches) mediate 31.1%, 57%, and 78% of apical oxalate transport, respectively. A1 mediates 30.9% (59.5% reduced protein abundance) of basolateral oxalate transport. A3 (78.7% reduced protein abundance) has no significant contribution to apical oxalate transport. In conclusion, most apical oxalate transport in C2 cells is mediated by A2+A6 (with no significant contribution from A3) while A1 contributes to basolateral oxalate transport. Thus, C2 cells can be used for screening/testing novel drugs targeting these transporters, which will have significant potential therapeutic implications for oxalate‐related disorders.

## 1 INTRODUCTION

Kidney stone disease remains a major health problem which imposes major clinical and economic burdens (Foster et al., 2006; Litwin, 2012; Scales Jr. et al., 2012; Thongprayoon et al., 2020). 70%–80% of kidney stones are composed of calcium oxalate (CaOx) and even small increases in urinary oxalate concentration significantly enhance CaOx supersaturation and the risk for stone formation (Curhan & Taylor, 2008; D'Costa et al., 2021; Robertson & Hughes, 1993). The recurrence rate is high, up to 50% and 80% in 5 and 10–20 years, respectively (Coe et al., 1977; Coe et al., 2005; Ljunghall, 1987). In addition to kidney stones, oxalate also potentially contributes to chronic kidney disease (CKD) and its progression, CKD‐ and end stage kidney disease (ESKD)‐associated cardiovascular diseases, compromised renal transplant outcomes, and progression of cyst growth in autosomal dominant polycystic kidney disease (ADPKD) (Bagnasco et al., 2009; Efe et al., 2019; Gulhan et al., 2015; Palsson et al., 2018; Puurunen et al., 2024; Torres et al., 2019; Waikar et al., 2019). Interestingly, elevated plasma oxalate in ESKD patients is associated with increased risk of a cardiovascular event and sudden cardiac death (Pfau et al., 2021). Therefore, besides preventing recurrent kidney stones, lowering plasma and/or urinary oxalate levels could potentially slow CKD progression and improve cardiovascular and renal transplant outcomes in CKD/ESKD patients and kidney transplant recipients, respectively.

The mammalian intestine plays an essential role in oxalate homeostasis by acting as a site for oxalate absorption and together with the kidney as sites for oxalate excretion (Abdelhalim et al., 2026; Hatch & Freel, 2005). Intestinal oxalate absorption is predominantly passive through the paracellular pathway, while secretion is active through the transcellular pathway (Abdelhalim et al., 2026; Freel et al., 2006; Hatch & Freel, 2005; Jiang et al., 2006; Knauf et al., 2011). The intestine's crucial role in overall oxalate homeostasis is shown by the following studies in relevant transporters belonging to the SLC26 family of anion exchangers: (1) SLC26A6 (A6) null mice have a critical defect in small intestinal transcellular oxalate secretion, leading to enhanced absorption of ingested oxalate, hyperoxalemia, hyperoxaluria, and CaOx kidney stones (Freel et al., 2006; Jiang et al., 2006). Importantly, *Oxalobacter formigenes (Of)*‐derived factors reduced urinary oxalate excretion in hyperoxaluric mice by enhancing transcellular distal colonic oxalate secretion via mechanisms involving A6 and SLC26A2 (A2) in human intestinal Caco2‐BBE (C2) cells (Arvans et al., 2017; Arvans et al., 2023). (2) SLC26A3 (A3) null mice have decreased transcellular ileal, cecal, and distal colonic oxalate absorption, as well as reduced (66%) urinary oxalate excretion (Freel et al., 2013), reflecting its critical role in transcellular intestinal oxalate absorption. Interestingly, the small molecule A3 inhibitor (DRA_inh_‐A270) decreased (by 70%) oxalate absorption in closed colonic loops and reduced urinary oxalate excretion following acute sodium oxalate loading in mice (Cil et al., 2022).

Strategies aimed at enhancing transcellular intestinal oxalate secretion and/or reducing transcellular intestinal oxalate absorption have great potential to lower both plasma and urinary oxalate levels. However, this requires detailed understanding of the involved oxalate transporters, including knowledge of their major regulatory mechanisms, in relevant intestinal cell lines to be used as reliable in vitro model systems to screen for potential novel therapeutics targeting these transporters and how they impact them. Using siRNA knockdown studies, we previously showed that A6 is the main apical oxalate transporter in C2 cells, followed by A2 (Arvans et al., 2017; Arvans et al., 2020; Arvans et al., 2023). The purpose of this study was to use siRNA knockdown to (1) Simultaneously (i.e., in the same batches of cells) silence A2, A6, and combined A2+A6 to test our hypothesis that A2+A6 mediates most (if not all) apical oxalate transport in C2 cells; (2) Assess the contribution of A3 to apical oxalate transport in C2 cells; (3) Assess the contribution of the anion exchanger SLC26A1 (A1) to basolateral oxalate transport in C2 cells. Transcellular intestinal oxalate secretion in vivo requires oxalate influx into the enterocyte from blood, and then its efflux from the luminal side (by A6 and potentially A2). A1 is an attractive candidate to serve as the basolateral oxalate transporter working in series with A6 as described under Discussion.

Our results show that (1) A2 (at 74% reduced mRNA expression), A6 (at 64% reduced total protein abundance), and A2+A6 mediate 31.1%, 57%, and 78% of apical oxalate transport in C2 cells; (2) A1 mediates 30.9% (at 59.5% reduced total protein abundance) of basolateral oxalate transport in C2 cells; (3) A3 (at 78.7% reduced total protein abundance) has no significant contribution to apical oxalate transport in C2 cells. These results support our hypothesis that most apical oxalate transport in C2 cells is mediated by A2+A6 and that these cells can be used as a reasonable in vitro model for novel drug screening and to define the underlying molecular mechanisms by which they regulate them.

## 2 MATERIALS AND METHODS

### 2.1 Cell culture

Human intestinal Caco2‐BBE (C2) cells were grown and maintained following our published methods (Amin et al., 2013; Hassan et al., 2012). Briefly, C2 were grown under standard conditions in DMEM High Glucose supplemented with 8%–10% fetal bovine serum (FBS), 50 U/mL penicillin, and 50 mg/mL streptomycin at 37°C in 5% CO_2_‐95% air. The C2 cells were obtained from Dr. Eugene Chang's laboratory (University of Chicago), which had previously obtained them from Dr. Mark Mooseker [Yale University; (Peterson & Mooseker, 1992); sex = male]. The C2 cells have been maintained in culture or as frozen stocks after being cultured since then. The oxalate flux and other studies described below were performed using confluent cells grown on 0.4‐μm Transwell membrane filters (Corning, cat. no. 3401) in 12‐mm inserts.

### 2.2 SLC26A1 (A1), SLC26A2 (A2), SLC26A3 (A3), and SLC26A6 (A6) siRNA knockdown in C2 cells

Using slight modification of a published protocol (Freel et al., 2009), we had previously utilized siRNA to knock down A2 and A6 expression in C2 cells (Arvans et al., 2017; Arvans et al., 2020; Arvans et al., 2023). Using the same protocol (including the same A2, A6, and negative control siRNAs), we similarly knocked down A2 and A6 expressions in C2 cells. Briefly, C2 cells were untransfected (UT) or transfected with the negative control siRNA (NC siRNA; Cat # AM4611‐3690528207, ThermoFisher Scientific) or the A6 specific siRNA (A6 siRNA; Cat # AM16708‐3690520878, ThermoFisher Scientific), or the A2 specific siRNA (A2 siRNA; Cat # AM16708‐3667241045, ThermoFisher Scientific), and the relevant studies were then performed 3 days after transfection. Of note is that the previously reported studies were performed 2–4 days after transfection (Arvans et al., 2017; Arvans et al., 2020; Arvans et al., 2023).

Human A1 and A3 Pre‐designed siRNAs (MedChemExpress = MCE) were used to knock down A1 and A3 expressions in C2 cells per manufacturer's protocol with slight modification. Briefly, C2 cells were seeded to be ~80%–90% confluent the next day at the time of transfection with the different siRNAs. Prior to transfection, complete media (containing FBS) were removed from the cells and replaced with serum‐free media. The apical side was filled with 400 μL of serum‐free media for A3 knockdown while the basolateral side was filled with 800 μL of serum‐free media for A1 knockdown. The transfection solution was prepared in serum‐free media by mixing the A1, A3, and NC siRNA (MCE; 0.5 μL in 50 μL) with SilentFect lipid reagent (Bio‐Rad Laboratories; cat. no. 1703361; 0.5 μL in 50 μL) at a 1:1 ratio (50 μL of each siRNA and SilentFect solutions). The mixtures (100 μL) were incubated for 30 min at room temperature and then added to the 400 μL (the final siRNA concentration is 20 nM) in the apical side and the cells were then incubated at 37°C in a CO₂ incubator. For knocking down A1 on the basolateral side, the procedure is the same except for doubling the volumes, but the final siRNA concentration remains the same (20 nM). The serum‐free media were replaced with complete growth media 4–6 h after transfection, and cells were maintained for an additional 3 days. Untransfected (UT) cells served as controls. Total RNA was harvested from these cells at 24‐, 48‐, and 72‐h post‐transfection to determine the optimal time point with regard to transfection efficiency. The highest reductions in A1 and A3 mRNA expression levels were observed at 24 and 48 h after transfection, respectively.

### 2.3 Real‐time quantitative PCR (RT‐qPCR)

To isolate total RNA from C2 cells, the cell monolayers were scraped into TRIzol reagent (Invitrogen, cat. no. 15596026). Briefly, the total RNA was extracted with chloroform, precipitated with isopropanol, and resuspended in nuclease‐free water. The extracted RNA was reverse transcribed into cDNA using the High‐Capacity cDNA Reverse Transcription Kit (Applied Biosystems, cat. no. 4374966). The amplification reaction was conducted using PowerUP SYBR Green master mix (Applied Biosystems, cat. no. 4367659) and cycled 40 times on a StepOnePlus real‐time PCR instrument (Applied Biosystems). Human A1 was amplified with gene‐specific primers (forward primer, 5′‐GGACTCCCTCAGCGTGCA AACA‐3′; reverse primer, 5′‐GCGTTAGGGAGGCGGTGCTTAG‐3′). Human A2 was amplified with gene‐specific primers (forward primer, 5=‐CGCGGGCGTTTACACTGGCT‐3=; reverse primer, 5=‐GTCCCTTTCCCCGCCCATCG‐3=). Human A3 was amplified with gene‐specific primers (forward primer, 5′‐GCTTCGGGATCGCAATGGTTGC‐3′; reverse primer, 5′‐TGGAGAGGGCAGTACTCCCAG‐3′). Human GAPDH was amplified as an internal control using gene‐specific primers (forward primer, 5′‐TCCCTGAGCTGAACGGGAAG‐3′; reverse primer, 5′‐GGAGGAGTGGGTGTCGCTGT‐3′). Relative A1, A2, and A3 mRNA expression levels were expressed as a percentage of UT normalized to GAPDH.

### 2.4 Western blot analysis

Western blotting was performed to assess A1, A3, and A6 total protein abundance following siRNA‐mediated knockdown. For A6, the procedure was similar to what we previously reported (Arvans et al., 2017) using the same anti‐A6 and anti‐GAPDH antibodies. For A1 and A3, C2 cells were lysed in Invitrogen™ Cell Extraction Buffer (Thermo Fisher Scientific, cat. no. FNN0011) according to the manufacturer's instructions, and total protein concentrations were determined using the Pierce™ BCA Protein Assay Kit (Thermo Scientific, cat. no. 23227). Equal amounts of protein (30 μg per lane) were mixed with Laemmli sample buffer containing DTT and loaded onto the SDS‐PAGE gel. The Precision Plus Protein™ Dual Xtra Prestained Protein Standards (Bio‐Rad, cat. no. 1610377) was used as the molecular weight marker. Following electrophoresis, proteins were transferred to Immobilon®‐P PVDF membranes (Millipore cat. no. IPVH00010) using standard wet‐transfer conditions. Membranes were blocked for 1 h at room temperature in 5% nonfat dry milk prepared in 1× phosphate‐buffered saline (PBS) containing 0.1% Tween‐20. Membranes were then incubated overnight at 4°C with rabbit anti‐A3 (Proteintech, cat. no. 87216‐1‐RR; 1:2000), rabbit anti‐A1 (Proteintech, cat. no. 26611‐1‐AP; 1:1000), or mouse monoclonal anti‐GAPDH (Santa Cruz Biotechnology, cat. no. sc‐32233; 1:1000). Following primary antibody incubation, membranes were washed three times with Tris‐buffered saline containing 0.1% Tween‐20 (TBST) and incubated for 1 h at room temperature with the corresponding HRP‐conjugated secondary antibody. A1 and A3 were detected using an HRP‐conjugated anti‐rabbit secondary antibody (Jackson ImmunoResearch Laboratories, cat. no. 711‐035‐152), whereas GAPDH was detected using an HRP‐conjugated anti‐mouse secondary antibody (Jackson ImmunoResearch Laboratories, cat. no. 715‐035‐150). Membranes were subsequently washed three times with TBST, and protein signals were developed using Pierce™ ECL Western Blotting Substrate (Thermo Scientific, cat. no. 32106) Western Blotting Substrate (Thermo Scientific, cat. no. 32106) according to the manufacturer's instructions. Chemiluminescent signals were captured using a ChemiDoc™ imaging system (Bio‐Rad). A1 and A3 were detected at the expected molecular weights of approximately 85 kDa, whereas GAPDH was detected at approximately 37 kDa. Specificities for the new antibodies for detection of A1 and A3 were validated using the following criteria: (1) The antibodies for A1 and A3 each consistently detected proteins at the appropriate respective molecular weights; (2) When A1 and A3 were knockdowned using siRNA these antibodies showed reduced total protein abundances that correlated with the observed decreases in mRNA expression levels; and (3) the negative control siRNA silencing experiments did not change mRNA expression levels nor total protein abundances of either A1 or A3 and were consistent with the untransfected cells. Band intensities were quantified by densitometric analysis using ImageJ, and A1, A3, and A6 abundance levels were normalized to GAPDH to evaluate the efficiency of siRNA‐mediated knockdown.

### 2.5 Radioactive flux

^14^C‐oxalate flux studies were performed to evaluate the impact of the siRNA‐mediated knockdown of A2, A3, and A6 on apical ^14^C‐oxalate uptake by C2 cells. Apical ^14^C‐oxalate flux studies in C2 cells were performed following our published methods (Amin et al., 2013; Hassan et al., 2012). Following aspiration of the culture medium, the cells were incubated in a Cl‐free solution (in mM: K‐gluconate = 130, glucose = 5, HEPES = 20, pH 7.4) containing 10–20 μM ^14^C‐oxalate (Vitrax, cat. no. VC 223) for 6 min. This 6‐min period was chosen based on our finding that it falls within the linear range of oxalate uptake by these cells. The uptake (influx) of ^14^C‐oxalate was terminated by 2–3 rapid washes of the cell monolayers with ice‐cold Cl‐free solution and the Transwells were then placed upside down and were allowed to dry for several minutes. Membrane filters containing the cells were cut from the support and placed into vials with scintillation fluid and radioactivity was measured by scintillation spectrometry following overnight solubilization. The impact of the siRNA‐mediated knockdown of A1 was assessed functionally by its effects on basolateral ^14^C‐oxalate uptake by C2 cells. The procedure is like what is described above with apical flux except it is done on the basolateral side.

### 2.6 Materials

Two separate batches of ^14^C‐oxalate were purchased from Vitrax (Placentia, CA). DMEM and FBS were obtained from Corning. Pen/Strep was obtained from Sigma Aldrich. Scintillation fluid (Bio‐Safe II Complete Counting Cocktail) was purchased from RPI. The rest of the supplies were described above under Materials and Methods.

### 2.7 Statistical analysis

Experimental data are presented as means ± S.E. Data were analyzed by one‐way analysis of variance (ANOVA) followed by Tukey post hoc test. *p* Values <0.05 were considered statistically significant.

## 3 RESULTS

### 3.1 Contributions of the anion exchangers SLC26A2 (A2) and SLC26A6 (A6) to apical intestinal oxalate transport in C2 cells

We previously knocked down A2 expression in C2 using siRNA to assess its role in the PKA‐ and *Of*‐derived peptides‐induced stimulation of oxalate transport in C2 cells and observed that about 23%–39% of baseline oxalate uptake in C2 cells is mediated by A2 (Arvans et al., 2020; Arvans et al., 2023). We also previously knocked down A6 expression in C2 using siRNA to assess its role in the PKA‐ and *Of*‐derived factors‐induced stimulation of oxalate transport by C2 cells and observed that about 49–65% of baseline oxalate uptake by C2 cells is mediated by A6 (Arvans et al., 2017; Arvans et al., 2020; Arvans et al., 2023). Another group also previously reported that A6 mediates about 50% of apical oxalate uptake by C2 cells (Freel et al., 2009). Our goal in this study is to compare the contribution of A2 versus A6 versus A2+A6 to oxalate transport in C2 cells using siRNA knockdown studies performed simultaneously in the same batches of C2 cells.

We used siRNA to knockdown A2 expression in C2 cells, and the knockdown efficiency was assessed by evaluating A2 mRNA expression level by RT‐qPCR. As seen in Figure 1a, the siRNA knockdown procedure significantly reduced (74%) A2 mRNA expression level 72 h post‐transfection. Importantly, the NC siRNA had no significant effect on A2 mRNA expression level, indicating that the observed reduction is not due to a general effect of the silencing procedure. Of note is that the observed reduction in A2 mRNA expression was similar 48–72 h post‐transfection, but less in the 24‐h time point. Importantly, we tested several commercial anti‐A2 antibodies and unfortunately, they did not work for immunoblotting. Therefore, we could not assess the impact of the siRNA knockdown on A2 total protein abundance. We next assessed the impact of the siRNA‐mediated knockdown of A2 on apical ^14^C‐oxalate uptake by C2 cells 72 h post‐transfection. A2 siRNA silencing significantly reduced (31.1%) apical ^14^C‐oxalate uptake by C2 cells (Figure 1b) while the NC siRNA had no significant effect, which is like what we previously reported (Arvans et al., 2020; Arvans et al., 2023).

**FIGURE 1:**
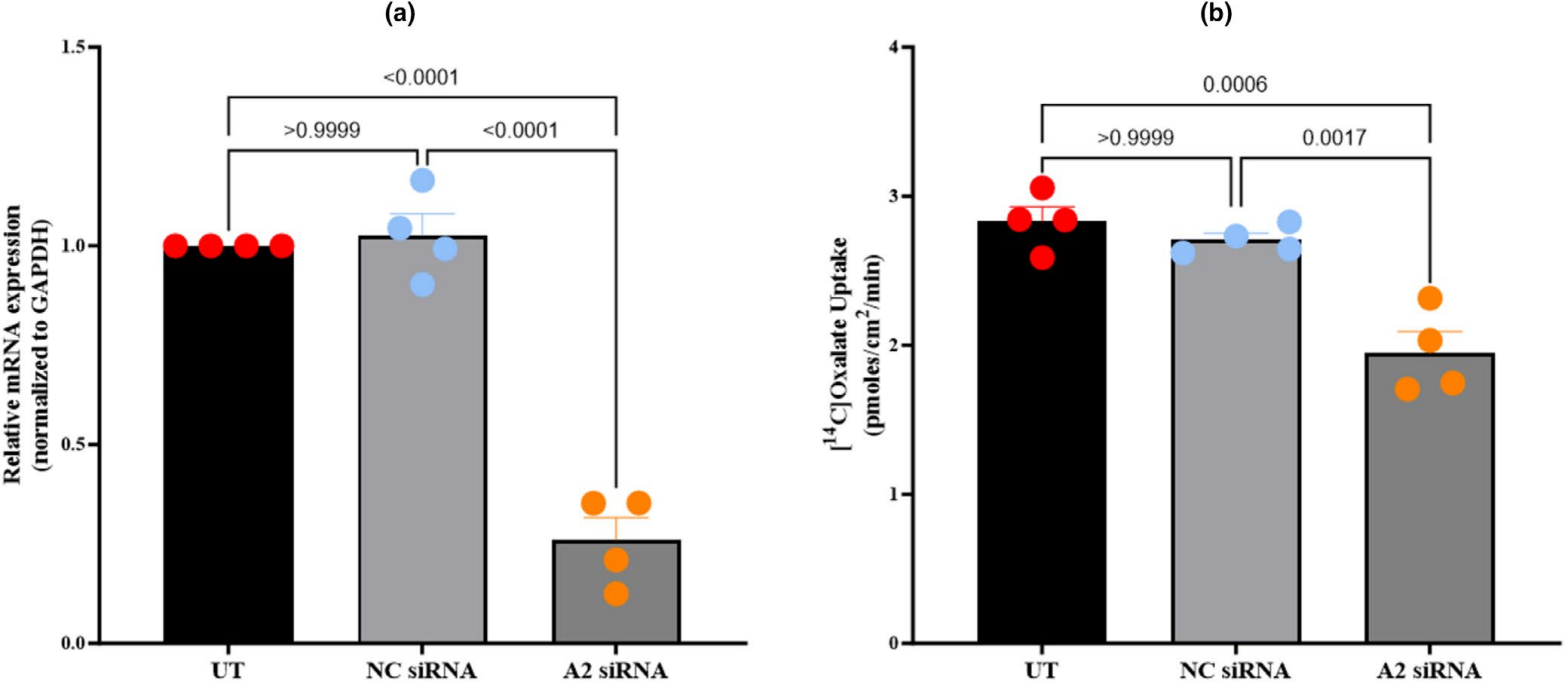
Effect of SLC26A2 (A2) siRNA knockdown on A2 mRNA expression (a) and apical ^14^C‐oxalate uptake (b) in Caco2‐BBE (C2) cells. (a) C2 cells were untransfected (UT), transfected with the negative control siRNA (NC siRNA), or transfected with the siRNA targeting A2 (A2 siRNA) and then total RNA was isolated for RT‐qPCR analysis. Values are means ± SE of 4 independent experiments, each of which was done in duplicate or triplicate. Relative A2 mRNA expression level was expressed as a percentage of UT normalized to GAPDH. siRNA knockdown of A2 significantly reduced its mRNA expression level (the *p* values are provided on the bar graphs; analyzed by ANOVA). (b) ^14^C‐oxalate uptake by UT, NC siRNA, and A2 siRNA C2 cells was measured as described in Methods. Values are means ± SE of 4 independent experiments, each of which was done in duplicate or triplicate. Silencing A2 significantly reduced apical oxalate transport by C2 cells (the *p* values are provided on the bar graphs; analyzed by ANOVA).

We similarly knocked down A6 expression in C2 cells, which led to significant reduction (64%) in A6 total protein abundance level 72 h post‐transfection (Figure 2a,b), which is similar to what we and another group had previously reported (Arvans et al., 2017; Freel et al., 2009). Worthy of mentioning is that we previously observed similar A6 knockdown efficiency in T84 cells using shRNA (Hassan et al., 2012). Equal protein loading is ensured by similar GAPDH abundance. Importantly, the siRNA silencing of A6 significantly reduced (57%) apical ^14^C‐oxalate uptake by C2 cells (Figure 3) while the NC siRNA had no significant effect, which is similar to what we and another group had previously reported (Arvans et al., 2017; Arvans et al., 2020; Arvans et al., 2023; Freel et al., 2009).

**FIGURE 2:**
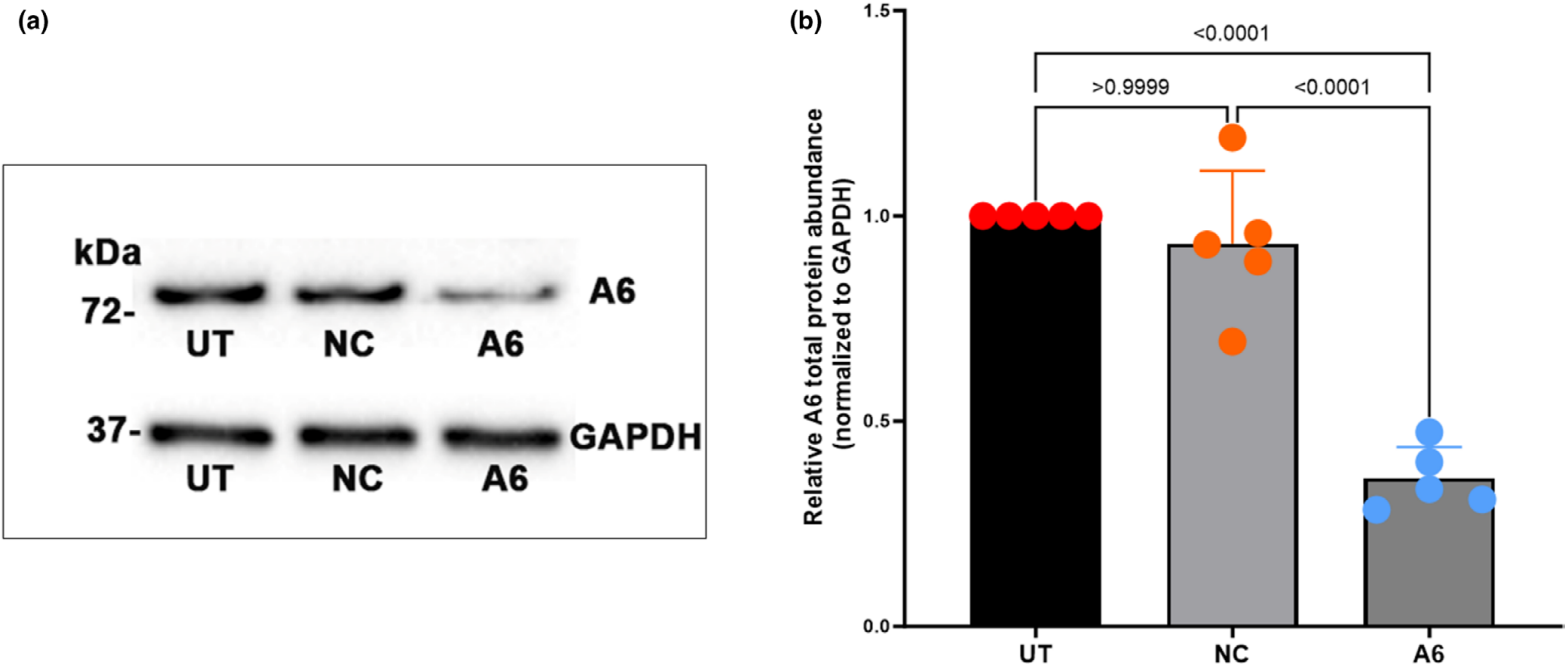
Effect of SLC26A6 (A6) siRNA knockdown on A6 total protein abundance. C2 cells were untransfected (UT), transfected with the negative control siRNA (NC), or transfected with the siRNA targeting A6 (A6), and then total protein was isolated for Western blot analysis. (a) A representative Western blot. (b) Band intensities were quantified by densitometric analysis using ImageJ, and A6 total protein abundance levels were normalized to GAPDH to evaluate the efficiency of siRNA‐mediated knockdown. Values are means ± SE of 5 independent experiments. The siRNA knockdown of A6 significantly reduced its total protein abundance level (the *p* values are provided on the bar graphs; analyzed by ANOVA).

**FIGURE 3:**
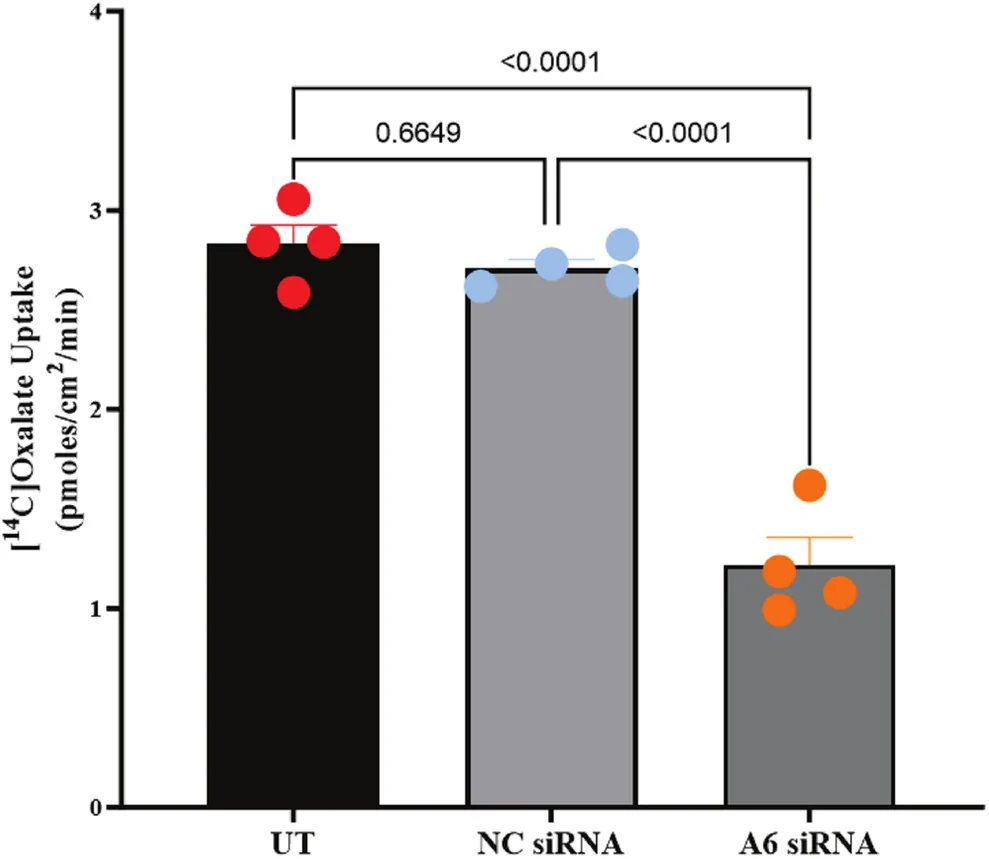
Effect of SLC26A6 (A6) siRNA knockdown on apical ^14^C‐oxalate uptake by Caco2‐BBE (C2) cells. ^14^C‐oxalate uptake by untransfected (UT), NC siRNA (transfected with the negative control siRNA), and A6 siRNA (transfected with the siRNA targeting A6) C2 cells was measured as described in Methods. Values are means ± SE of 4 independent experiments each of which was done in duplicate or triplicate. Silencing A6 significantly reduced apical oxalate transport by C2 cells (the *p* values are provided on the bar graphs; analyzed by ANOVA).

We hypothesized that most (if not all) of apical oxalate transport in C2 cells is mediated by A2+A6, with A6 having the largest contribution. To test this hypothesis, we similarly knocked down both A2 and A6 (A2+A6 double knockdown) in the same batches of C2 cells used for individually knocking down A2 or A6. A2+A6 combined siRNA silencing significantly reduced (78%) apical ^14^C‐oxalate uptake by C2 cells (Figure 4) while the NC siRNA had no significant effect. Importantly, we verified in pilot studies that the knockdown efficiencies in the monolayers used for the combined A2+A6 knockdown are similar to what we observed in the monolayers used for individual A2 or A6 knockdown. These results support our hypothesis that most apical oxalate transport in C2 cells is mediated by A2+A6.

**FIGURE 4:**
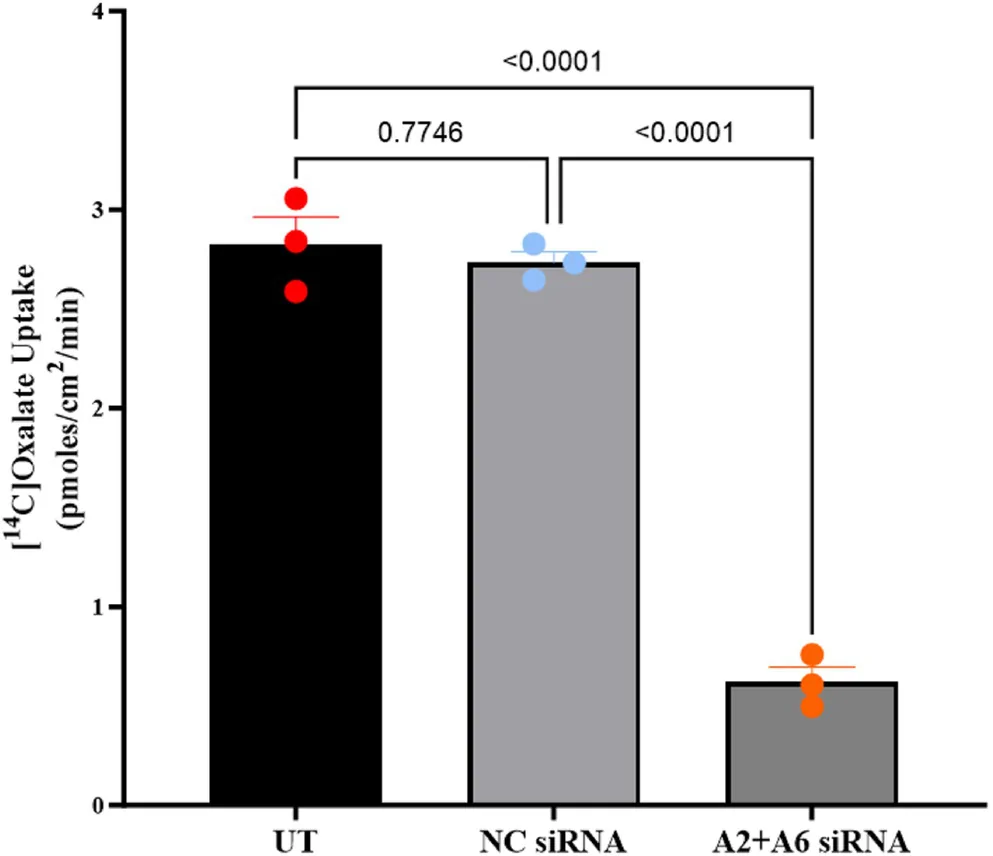
Effect of combined SLC26A2 (A2) and SLC26A6 (A6) siRNA knockdown on apical ^14^C‐oxalate uptake by Caco2‐BBE (C2) cells. ^14^C‐oxalate uptake by untransfected (UT), NC siRNA (transfected with the negative control siRNA), and A2+A6 siRNA (transfected with the siRNAs targeting A2 and A6) C2 cells was measured as described in Methods. Values are means ± SE of 3 independent experiments each of which was done in duplicate or triplicate. Silencing A2+A6 significantly reduced apical oxalate transport by C2 cells (the *p* values are provided on the bar graphs; analyzed by ANOVA).

### 3.2 Contribution of the anion exchanger SLC26A1 (A1) to basolateral intestinal oxalate transport in C2 cells

Transcellular intestinal oxalate secretion in vivo requires oxalate influx into the enterocyte from blood, and then its efflux from the luminal side where it can then be excreted in the stool and/or metabolized by gut oxalate‐degrading bacteria. A6 plays a crucial role in mouse small intestinal transcellular oxalate secretion (Freel et al., 2006; Jiang et al., 2006), thereby defending against hyperoxalemia, hyperoxaluria, and related kidney stones. The basolateral oxalate transporter working in series with A6 to complete the transcellular oxalate secretion remains unknown. A1 protein was reported in the basolateral aspects of the mouse distal ileum, cecum, and proximal colon, where it likely plays an important role in distal ileal, cecal, and proximal colonic oxalate transport (Dawson et al., 2010). Therefore, A1 is an attractive candidate to mediate this process. Thus, we investigated the contribution of A1 to basolateral intestinal oxalate transport by knocking down its expression in C2 cells using siRNA.

As seen in Figure 5a, siRNA silencing of A1 significantly reduced (68.4%) its mRNA expression level 24 h post‐transfection (less reduction was observed at 48–72 h post‐transfection), while NC siRNA had no significant effect on A1 mRNA expression level. mRNA‐protein discordance is common; thus, we verified that the observed reduction in A1 mRNA expression is translated into decreased A1 total protein abundance using immunoblotting. The siRNA knockdown significantly reduced (59.5%) A1 total protein abundance (Figure 5b,c), while the NC siRNA had no significant effect. Equal protein loading is ensured by similar GAPDH abundance. The functional relevance of the siRNA‐mediated A1 knockdown was assessed by evaluating its impact on basolateral ^14^C‐oxalate uptake in C2 cells 24 h post‐transfection. A1 siRNA silencing significantly reduced (30.9%) basolateral ^14^C‐oxalate uptake by C2 cells (Figure 6), while the NC siRNA had no significant effect. This finding indicates that A1 significantly contributes to basolateral intestinal oxalate transport in C2 cells.

**FIGURE 5:**
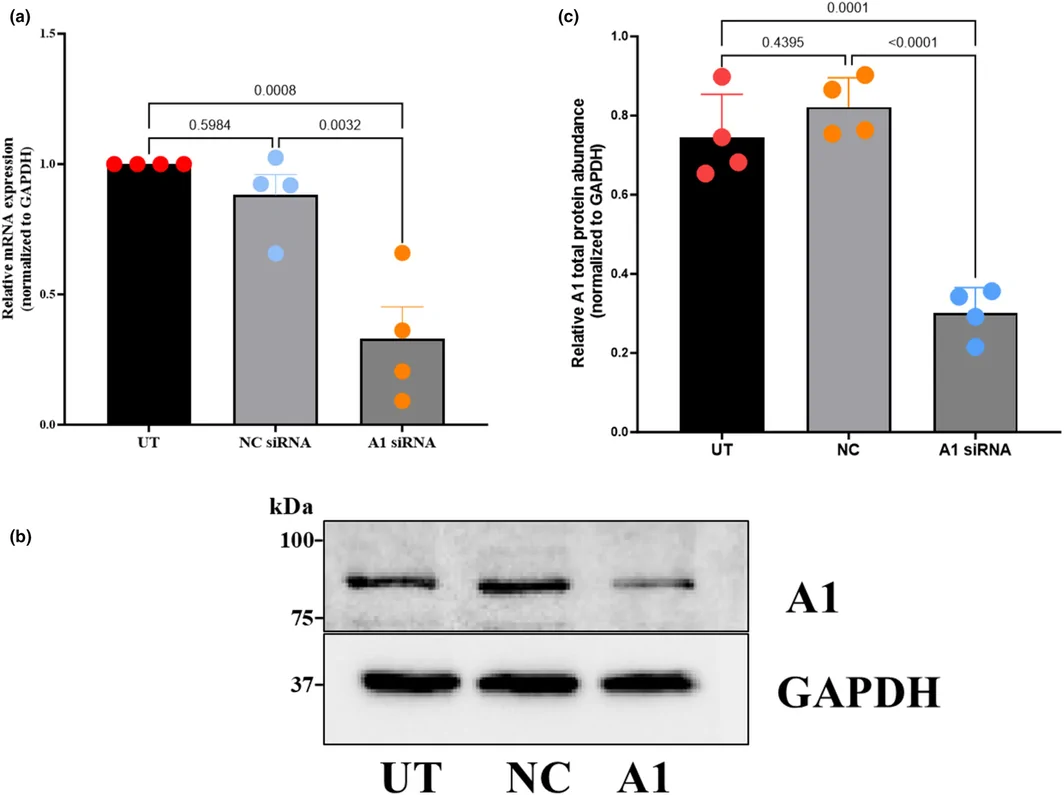
Effect of SLC26A1 (A1) siRNA knockdown on A1 mRNA expression (a) and total protein abundance (b and c) in Caco2‐BBE (C2) cells. (a) C2 cells were untransfected (UT), transfected with the negative control siRNA (NC siRNA), or transfected with the siRNA targeting A1 (A1 siRNA), and then total RNA was isolated for RT‐qPCR analysis. Values are means ± SE of 4 independent experiments, each of which was done in duplicate or triplicate. Relative A1 mRNA expression level was expressed as a percentage of UT normalized to GAPDH. siRNA knockdown of A1 significantly reduced its mRNA expression level (the *p* values are provided on the bar graphs; analyzed by ANOVA). (b) A representative Western blot. C2 cells were untransfected (UT), transfected with the negative control siRNA (NC), or transfected with the siRNA targeting A1 (A1), and then total protein was isolated for Western blot analysis. (c) Band intensities were quantified by densitometric analysis using ImageJ, and A1 abundance levels were normalized to GAPDH to evaluate the efficiency of siRNA‐mediated knockdown. Values are means ± SE of 4 independent experiments. siRNA knockdown of A1 significantly reduced its total protein abundance level (the *p* values are provided on the bar graphs; analyzed by ANOVA).

**FIGURE 6:**
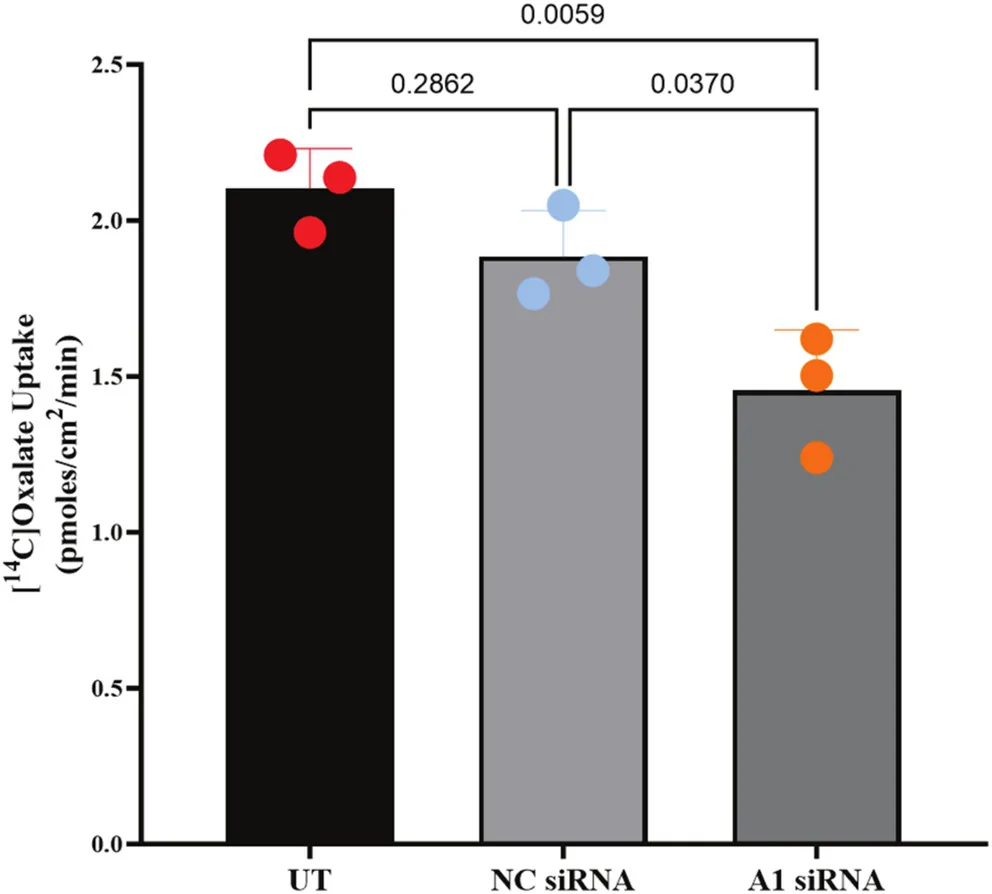
Effect of SLC26A1 (A1) siRNA knockdown on basolateral ^14^C‐oxalate uptake by Caco2‐BBE (C2) cells. ^14^C‐oxalate uptake by untransfected (UT), NC siRNA (transfected with the negative control siRNA), and A1 siRNA (transfected with the siRNA targeting A1) C2 cells was measured as described in Methods. Values are means ± SE of 3 independent experiments, each of which was done in duplicate or triplicate. Silencing A1 significantly reduced basolateral oxalate transport by C2 cells (the *p* values are provided on the bar graphs; analyzed by ANOVA).

### 3.3 Contribution of the anion exchanger SLC26A3 (A3) to apical intestinal oxalate transport in C2 cells

Mice lacking A3 have decreased transcellular ileal, cecal, and distal colonic oxalate absorption, as well as reduced (66%) urinary oxalate excretion (Freel et al., 2013), reflecting the importance of A3 in transcellular intestinal oxalate absorption. However, in contrast to the observed robust Cl^−^‐ HCO_3_^−^ exchange, human A3 transports oxalate poorly when expressed in Xenopus oocytes (Chernova et al., 2003), strongly suggesting that Cl^−^‐ HCO_3_^−^ exchange is the main physiological function of A3. Since C2 cells better resemble the native intestinal epithelium compared to Xenopus oocytes, we used a similar approach to knockdown A3 expression to assess its contribution to apical intestinal oxalate transport in C2 cells.

siRNA knockdown of A3 significantly reduced (76.5%) its mRNA expression level 48 h post‐transfection, while NC siRNA had no significant effect (Figure 7a). We used immunoblotting to verify that the observed reduction in A3 mRNA expression is indeed translated into decreased A3 total protein abundance. The siRNA knockdown significantly reduced (78.7%) A3 total protein abundance (Figure 7b,c), while the NC siRNA had no significant effect. Equal loading is ensured by similar GAPDH abundance. We utilized flux studies to assess the functional significance of A3 siRNA‐mediated knockdown on apical ^14^C‐oxalate uptake in C2 cells 48 h post‐transfection. A3 siRNA silencing led to a statistically insignificant reduction (13%) in apical ^14^C‐oxalate uptake by C2 cells (Figure 8). The NC siRNA also had no significant effect on apical ^14^C‐oxalate uptake by C2 cells. These results indicate that A3 does not play an important role in apical intestinal oxalate transport in C2 cells.

**FIGURE 7:**
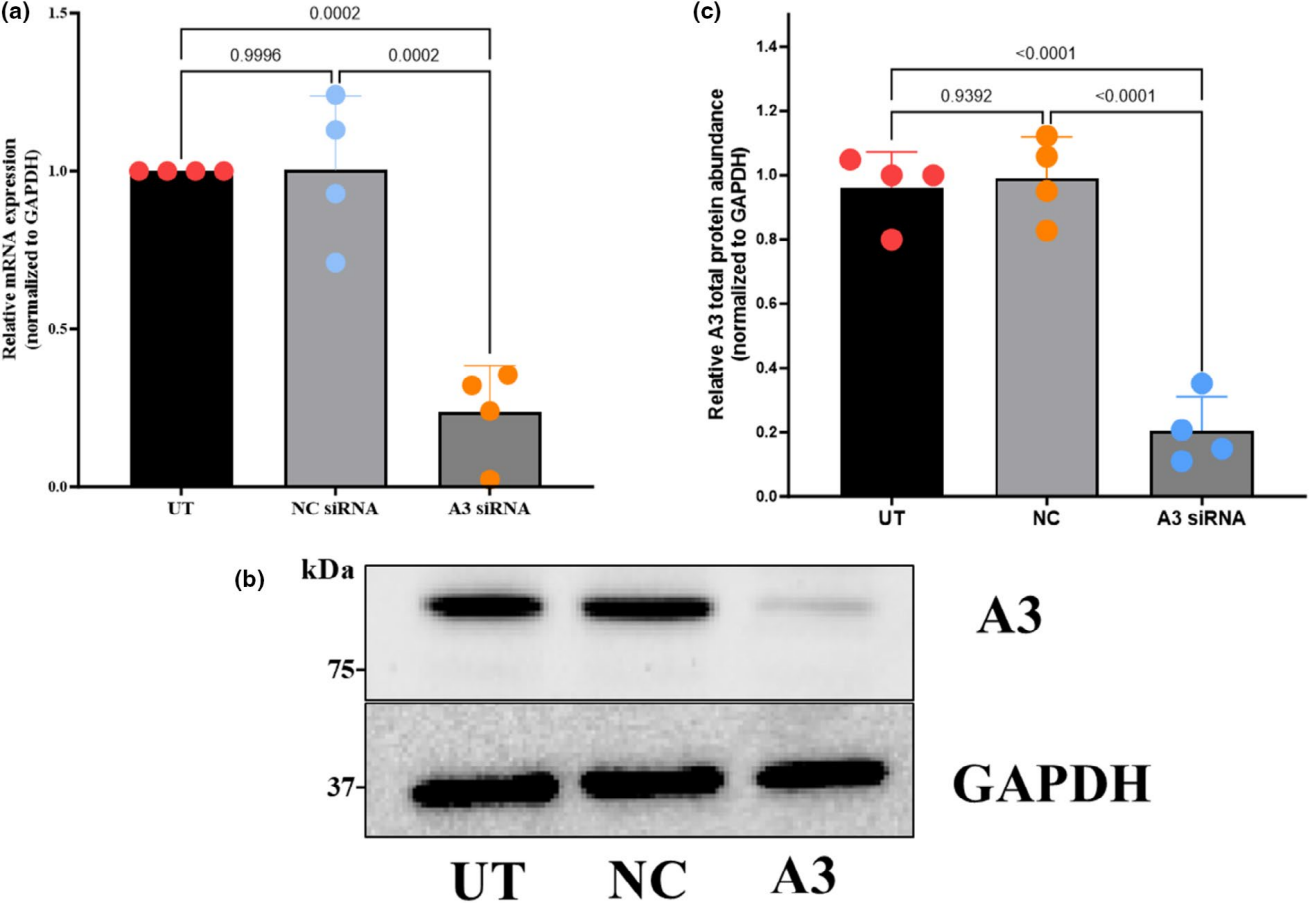
Effect of SLC26A3 (A3) siRNA knockdown on A3 mRNA expression (a) and total protein abundance (b and c) in Caco2‐BBE (C2) cells. (a) C2 cells were untransfected (UT), transfected with the negative control siRNA (NC siRNA), or transfected with the siRNA targeting A3 (A3 siRNA), and then total RNA was isolated for RT‐qPCR analysis. Values are means ± SE of 4 independent experiments each of which was done in duplicate or triplicate. Relative A3 mRNA expression level was expressed as a percentage of UT normalized to GAPDH. The siRNA knockdown of A3 significantly reduced its mRNA expression level (the *p* values are provided on the bar graphs; analyzed by ANOVA). (b) A representative Western blot. C2 cells were untransfected (UT), transfected with the negative control siRNA (NC), or transfected with the siRNA targeting A3 (A3), and then total protein was isolated for Western blot analysis. (c) Band intensities were quantified by densitometric analysis using ImageJ, and A3 expression levels were normalized to GAPDH to evaluate the efficiency of siRNA‐mediated knockdown. Values are means ± SE of 4 independent experiments. siRNA knockdown of A3 significantly reduced its total protein abundance level (the *p* values are provided on the bar graphs; analyzed by ANOVA).

**FIGURE 8:**
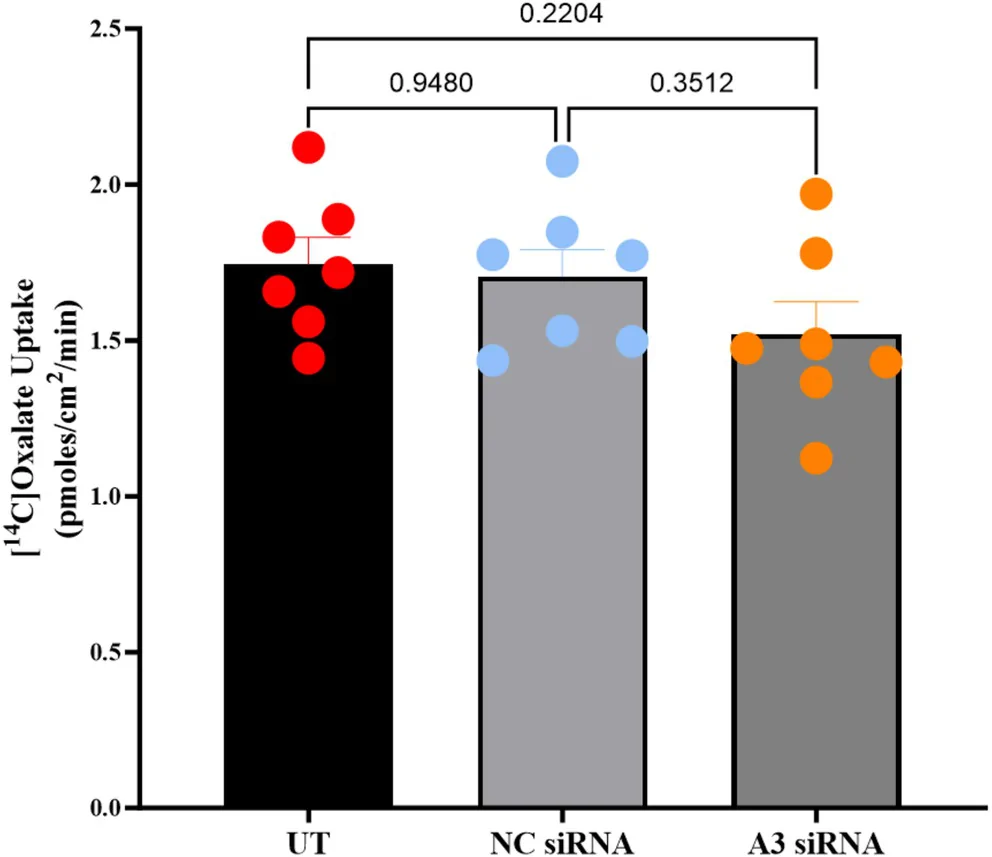
Effect of SLC26A3 (A3) siRNA knockdown on apical ^14^C‐oxalate uptake by Caco2‐BBE (C2) cells. ^14^C‐oxalate uptake by untransfected (UT), NC siRNA (transfected with the negative control siRNA), and A3 siRNA (transfected with the siRNA targeting A3) C2 cells was measured as described in Methods. Values are means ± SE of 7 independent experiments, each of which was done in duplicate or triplicate. Silencing A3 had no significant effect on apical oxalate transport by C2 cells.

## 4 DISCUSSION

This study used siRNA‐mediated knockdown and flux functional assays to examine the relative contributions of four SLC26 anion exchangers (A1‐3, and 6) to apical and basolateral oxalate transport in C2 cells. Knockdown of A2, A6, and combined A2+A6 reduced apical oxalate transport by 31.1%, 57%, and 78%, respectively. These findings support our hypothesis that most apical oxalate transport in C2 cells is mediated by A2 and A6. Similarly, A1 knockdown (at 59.5% decreased total protein abundance) resulted in a 30.9% reduction in basolateral oxalate transport, providing direct evidence for A1 at the basolateral entry step into enterocytes. However, A3 knockdown had no significant effect on apical oxalate transport despite 78.7% reduced total protein abundance.

The predominant effect of A6 siRNA silencing is consistent with our previous studies in C2 and T84 cells showing that A6 mediates about 49%–65% of apical oxalate transport (Arvans et al., 2017; Arvans et al., 2020; Arvans et al., 2023; Freel et al., 2009; Hassan et al., 2012). These findings are supported by the defective transcellular intestinal oxalate secretion in A6 null mice, leading to the observed phenotype of hyperoxalemia, hyperoxaluria, and calcium oxalate nephrolithiasis (Freel et al., 2006; Jiang et al., 2006). We previously identified *Of*‐derived factors secreted in its culture conditioned medium (CM) that stimulate (>2.4‐fold) oxalate transport in C2 cells through A6 (Arvans et al., 2017) and A2 (unpublished data). The CM‐induced stimulation in C2 cells translated into an in vivo effect since CM rectal administration reduced (>32.5%) urinary oxalate excretion in hyperoxaluric mice by stimulating (>42%) transcellular distal colonic oxalate secretion (Arvans et al., 2017). We also observed that intestinal oxalate transport in C2 cells by two other regulatory factors translated into similar regulation in vivo in mice (unpublished data). Based on these observations, we believe it is reasonable to say that C2 cells resemble the native intestinal epithelium from a gut oxalate transport perspective, with these cells modeling transporter capacity and not necessarily the in vivo dominance. Since C2 cells resemble the native intestinal epithelium, A6 is expected to be the major oxalate transporter mediating transcellular intestinal oxalate secretion in vivo in humans as observed in mice (Freel et al., 2006; Jiang et al., 2006). A6 is highly expressed in mouse small intestine, with much lower expression in the colon (Wang et al., 2002). However, its role in transcellular colonic oxalate secretion remains unknown. Interestingly, A6 expression is >1.8‐fold higher in human colon compared to human small intestine (Fagerberg et al., 2014), suggesting that A6 likely plays an important role in human transcellular colonic oxalate secretion in vivo. Our studies in human colon‐derived C2 and T84 cells (Arvans et al., 2017; Arvans et al., 2020; Hassan et al., 2012) and another study (Freel et al., 2009) and human colonic organoids (Arvans et al., 2023) support this notion.

A2 is apically expressed in enterocytes (colon > small intestine) (Alper & Sharma, 2013; Haila et al., 2001; Satoh et al., 1998) and is capable of oxalate transport when expressed in heterologous systems (Heneghan et al., 2010). However, its role in intestinal oxalate transport in vivo remains unknown. A2 mediates 31.1% of apical oxalate transport in C2 cells in this study, which is like our previous reports (Arvans et al., 2020; Arvans et al., 2023). This provides further support for its important contribution to apical intestinal oxalate transport in C2 cells, and likely a similar role in transcellular intestinal oxalate secretion in vivo in humans. Since A2, A6, and combined A2+A6 siRNA knockdown studies were simultaneously done in the same C2 batches, it is tempting to speculate that A6 mediates about 65%–75% of the total oxalate transport in these cells, while A2 likely mediates the remaining 25%–35%. A2 null mice are not available due to embryonic lethality, but A2 knock‐in mice with a loss‐of‐function mutation (A386V) showed a phenotype that closely recapitulates human diastrophic dysplasia (Forlino et al., 2005). Expression of this mutation (A386V) in Xenopus oocytes led to ~50% reduction in oxalate uptake (Heneghan et al., 2010), supporting a potentially important role for A2 in oxalate transport.

Transcellular intestinal oxalate secretion in vivo requires oxalate influx into the enterocyte from blood and then its efflux from the luminal side. A1 is an attractive candidate to serve as the basolateral oxalate transporter working in series with A6, given its reported localization and capability to transport oxalate (Freel & Hatch, 2012; Lee et al., 2003; Regeer et al., 2003). Our finding that A1 mediates 30.9% of basolateral oxalate transport in C2 cells at 59.5% reduced protein abundance highlights the potential importance of A1 to basolateral intestinal oxalate transport. Our results are in line with the hyperoxalemia observed in A1 null mice (on a mixed 129/JSv × CD1 background), which is likely due to decreased intestinal oxalate secretion since these mice have significantly reduced oxalate uptake by distal ileal, cecal (including lower cecal oxalate content), and proximal colonic basolateral membrane vesicles (Dawson et al., 2010). A1 null mice also have hyperoxaluria and calcium oxalate kidney stones (Dawson et al., 2010), which fits with clinical association studies linking A1 variants with nephrolithiasis susceptibility (Markovich, 2011; Soleimani, 2013; Xie et al., 2002). Surprisingly, A1 null mice have no hyperoxalemia or hyperoxaluria following transfer to a C57BL background, with A1 playing no role in duodenal, distal ileal, and distal colonic oxalate secretion (Ko et al., 2012; Whittamore et al., 2019). Differences in the genetic background might potentially explain the observed phenotype difference. It is also possible that dysregulation, rather than genetic defects, has a greater effect on gut oxalate transport and therefore urine oxalate. Together, A1 likely contributes to distal ileal, cecal, proximal colonic, and/or distal colonic oxalate transport.

Transcellular intestinal oxalate absorption requires luminal oxalate influx into the enterocyte, and then its efflux from the basolateral side into the blood. Studies in A3 null mice support the importance of A3 in transcellular intestinal oxalate absorption (Freel et al., 2013). Our results in C2 cells do not support an important role for A3 in apical intestinal oxalate transport, which is in line with previous two in vitro studies showing that human A3 transports oxalate poorly even at very high oxalate concentrations (Chernova et al., 2003; Wasiluk et al., 2019). This is in contrast to the in vivo data in A3 null mice (Freel et al., 2013) and the reported effects of the small molecule A3 inhibitor (DRA_inh_‐A270) on urinary oxalate excretion in mice (Cil et al., 2022). Potential explanations for this discrepancy include (1) Potential differences in transporter behavior in the in vitro setting compared to the in vivo environment, where A3 transport activity (including its affinity to oxalate) could be modulated by other regulatory factors (e.g., microbiota, dietary factors, etc.); (2) The prevailing in vivo electrochemical gradients and likely preserved interactions among multiple transport pathways. Since the magnitude and direction of counter‐ion driver gradients impact vectorial intestinal oxalate transport, complemented with the observation that human A3 transports oxalate poorly in vitro, it is possible that A3 deletion results in loss of counter‐ion driver gradients, leading to reduced transcellular intestinal oxalate absorption by unknown oxalate transporter(s), rather than being directly mediated by A3.

A2 and A6 are attractive potential novel therapeutic targets for enhancing transcellular intestinal oxalate secretion. We had previously used C2 cells to show (1) *Of*‐derived factors stimulate oxalate transport via PKA activation and A2 and A6 (Arvans et al., 2017; Arvans et al., 2020; Arvans et al., 2023); (2) Direct PKA stimulates oxalate transport in C2 through A2 and A6 (including enhancing A6 surface protein expression) (Arvans et al., 2020). Our study provides further support for using C2 cells as a valid human intestinal model to investigate the molecular mechanism regulating A2 and A6, including screening and testing the therapeutic effects of novel agents targeting A2 and/or A6. It also establishes them as a reasonable human intestinal model to study the same for A1, since its activation has the potential to enhance transepithelial oxalate secretion. Of interest in this regard is that we identified positive hits as potential A6 activators upon a virtual screen of a large compounds' library, with several hits stimulating apical oxalate uptake in C2 cells by >2‐fold (unpublished data).

A limitation of this study is that while we were able to confirm the impact of the siRNA knockdown on A1, A3, and A6 total protein abundance, we could not do so for A2 because none of the tested antibodies yielded reliable signals. However, we think it is reasonable to consider the observed reduced functional oxalate transport as indirect evidence that the decreased A2 mRNA expression was translated into some degree of reduced total A2 protein abundance.

In summary, we have confirmed that A2 and A6 account for most apical oxalate transport in C2 cells, with a considerably greater contribution being made by A6. We also showed that A1 significantly contributes to basolateral intestinal oxalate transport in C2 cells, while A3 has no significant contribution to apical intestinal oxalate transport in C2 cells. In addition to providing further support for using C2 cells as a valid human intestinal model for investigating A2 and A6 regulatory mechanisms, these results also establish them as a reasonable model to study A1 regulation. This will enable the screening and testing of novel drugs (including small molecule activators and/or positive allosteric modulators) for A1, A2, and/or A6, which will have significant therapeutic potential for hyperoxaluria, hyperoxalemia, and related disorders.

## AUTHOR CONTRIBUTIONS

**Hatim A. Hassan:** Conceptualization; data curation; formal analysis; funding acquisition; investigation; methodology; project administration; resources; supervision; validation. **Khalid A. Abdelhalim:** Data curation; formal analysis; methodology. **Yanzhe Wang:** Data curation. **Donna Arvans:** Data curation; formal analysis. **Mohamed Elfatih H. Bashir:** Data curation. Khalid A. Abdelhalim, Donna Arvans, and Yanzhe Wang contributed equally to this work.

## FUNDING INFORMATION

This work was largely supported by National Institutes of Health Grant DK121204 (H.A.H.) and some internal funding.

## CONFLICT OF INTEREST STATEMENT

Hatim A. Hassan, MD, PhD, co‐founder and Chief Scientific Officer for Oxalo Therapeutics. Dr. Hassan owns patents and shares in Oxalo Therapeutics, but the company had no role in the study design, data collection and analysis, decision to publish, or preparation of the manuscript.

## ETHICS STATEMENT

The study did not involve human subjects or animals and therefore there was no need for IRB approval.

## Data Availability

Data will be made available upon reasonable request.
